# Use of Simultaneous Nasogastric and Nasojejunal Tubes for Proximal Intestinal Atresias - A Preliminary Report

**DOI:** 10.21699/jns.v6i2.528

**Published:** 2017-04-15

**Authors:** Enono Yhoshu, JK Mahajan

**Affiliations:** Department of Pediatric Surgery, Postgraduate Institute of Medical Education and Research, Chandigarh India 160012

**Dear Sir**

Meeting the caloric needs of the baby in surgically repaired proximal intestinal atresias is a challenge. Total parenteral nutrition (TPN) is not readily available everywhere, especially in the resource crunched set-ups, besides sepsis being a prohibitive accompaniment of TPN. Technical difficulties of performing feeding jejunostomy in a small baby, along with added complications of a second surgical procedure deter its use for enteral feeds. We describe novel use of two nasal tubes, one in stomach for drainage and other being a trans-anastomotic nasojejunal tube for enteral feeding.

Over a period of 6 months, 6 neonates underwent repair for duodenal (4 cases) and proximal jejunal atresia (2 cases) in one Pediatric surgical unit. Each neonate was inserted two tubes, one through each nostril. Intraoperatively, one tube was manipulated across the anastomosis, while the other tube remained in the stomach. The atresias were repaired in the usual manner. Postoperatively, feeding was instituted through the nasojejunal tube, usually on 3rd or 4th post-operative day based on the abdominal condition of the baby. A contrast study was done through the nasogastric tube on 10th to 12th postoperative day and none of the patients showed anastomotic leak. Once patency of the anastomosis was confirmed, the nasogastric tube feeds were commenced. Gradually, the nasojejunal tube feeds were replaced by the nasogastric feeds. As the baby started tolerating feeds, both the tubes were removed. Total hospital stay ranged from 10 days to 4 weeks for 4 patients. None of the patients had any complications with the use of twin tubes. Two tubes in the nostrils caused no discomfort to the baby and also had no effect on respiration as monitored by the pulse oximetry. Other 2 patients expired after surgery; one was a case of multiple anomalies- tracheoesophageal fistula with esophageal atresia, anovestibular fistula, cleft palate and complex congenital heart disease and the other was a case of jejunal atresia, type-4 (apple peel) and died of sepsis.

Early feeding in any surgical case is of utmost importance for wound healing and general well-being of the patient. In intestinal atresias, trans-anastomotic or a feeding jejunostomy beyond the anastomosis, parenteral nutrition and gastrostomy have all been used with variable success.[1] Complications of the nasojejunal tubes such as duodenal and jejunal perforations, changes in bowel flora, abdominal distension, vomiting and diarrhoea have been reported in the past.[2] None of the above complications were encountered in our patients. Although intraoperative placement of tubes through separate enterotomy provides access for enteral nutrition, but it is so with the added disadvantage of risk of leaks, inadvertent tube removal, peristomal infection, bleeding and hematoma, ulceration, gastric outlet obstruction and perforation.[2] Trans-anastomotic tube via the nasal route avoids additional surgery. However, there are concerns and objections about neonates being obligate nasal breathers.[3,4] During the act of breast feeding, larynx elevates to enable the epiglottis to slide up behind the soft palate locking the larynx into nasopharynx. This allows the infant to both swallow and breathe at the same time.[4,5] However, because of smaller oral airway, larger volume of tongue and close proximity of epiglottis with soft palate, the babies prefer to breathe through their nose rather than their mouths. The neonates, though, preferential nasal breathers, will adapt to mouth breathing given the need. Hence use of double tubes via both nares in proximal intestinal atresias in neonates is safe and well tolerated as well as allows for early institution of enteral feeds.

## Footnotes

**Source of Support:** Nil

**Conflict of Interest:** None

## References

[1] Chen QJ, Gao ZG, Tou JF, Qian YZ, Li MJ, Xiong QX, et al. Congenital duodenal obstruction in neonates: a decade's experience from one center. World J Pediatr. 2014;10:238-44. 10.1007/s12519-014-0499-4 25124975

[2] Sun SC, Samuels S, Lee J, Marquis J. Duodenal perforation: A rare complication of neonatal nasojejunal tube feeding. Pediatrics 1975; 85:371-5. 806882

[3] Bergeson PS, Shaw JC. Are infants really obligatory nasal breathers? Clin Pediatr. 2001; 40:567-9. 10.1177/000992280104001006 11681824

[4] Lau C. Development of suck and swallow mechanisms in infants. Ann Nutr Metab. 2015; 66:7-14. 10.1159/000381361 PMC4530609 26226992

[5] Sakalidis VS, Kent JC, Garbin CP, Hepworth AR, Hartmann PE, Geddes DT. Longitudinal changes in suck-swallow-breathe, oxygen saturation, and heart rate patterns in term breastfeeding infants. J Hum Lact. 2013; 29:236-45. 10.1177/0890334412474864 23492760

